# Behavioural and Cognitive Effects of Cerebrovascular Diseases

**DOI:** 10.1155/2018/7516032

**Published:** 2018-04-15

**Authors:** Mayowa Owolabi, Akin Ojagbemi, Raj Kalaria, Fred Stephen Sarfo, Rufus Akinyemi

**Affiliations:** ^1^Department of Medicine, University of Ibadan, Ibadan, Nigeria; ^2^Department of Psychiatry, University of Ibadan, Ibadan, Nigeria; ^3^Institute of Neuroscience, Newcastle University, Campus for Ageing and Vitality, Newcastle upon Tyne NE4 5PL, UK; ^4^Department of Medicine, Kwame Nkrumah University of Science and Technology, Kumasi, Ghana; ^5^Institute of Advanced Medical Research and Training, University of Ibadan, Ibadan, Nigeria

Cerebrovascular diseases encompass a wide range of conditions that interfere with normal cerebral circulation mostly by causing changes in the integrity of relevant blood vessels, blood components, and hemodynamics. They include several subclinical and clinically significant causes of vascular brain injuries including intracranial atherosclerosis, aneurysms, vasculitis, vascular spasm, vascular malformations, chronic cerebral hypoperfusion, infarction, and haemorrhages. These conditions are associated with high disease burden worldwide and are now among the leading causes of disease in low- and middle-income countries (LMICs) undergoing socioeconomic transition. Recent advances in the management of cerebrovascular diseases have led to reduction in mortality rates. The impact of this progress on behavioural and cognitive outcomes of survivors remains unclear.

In some LMICs, stroke due to cerebral infarction or hemorrhage has an annual incidence rate that reaches up to 316 per 100,000 [1]. The projection for cerebrovascular diseases in LMICs is that of a continuous and steep rise. This is in sharp contrast to the declining incidence rates in high-income countries (HIC). Several studies report that vascular risk factors and cerebral infarcts boost risk for cognitive impairment, dementia, depression, and anxiety [2–4]. As such, the high and increasing burden of cerebrovascular diseases in LMICs is concomitant with an increase in behavioural and cognitive defects. Individually and collectively, poststroke depression, anxiety, and dementia constitute a major public health problem with substantial personal, social, and financial burden [5].

This issue of behavioural neurology provides insight into the behavioural and cognitive sequelae of cerebrovascular diseases, especially as expressed in survivors from LMICs where the burden is escalating and the resources to mitigate the rise is disproportionately low. The call for papers for the issue came just in time for the first World Federation of Neurorehabilitation (WFNR) Conference in East, West, and Central Africa, themed “Neurorehabilitation in Africa: Adaptations and Innovations.” This maiden conference of the WFNR was in collaboration with **t**he Blossom Medical Centre, Nigeria (a WFNR affiliated Institute), and the College of Medicine, University of Ibadan. The conference was a forum for the exchange and integration of innovative adaptive technologies in neurorehabilitation for improving the lives of patients across East, West, and Central Africa, as well as an avenue to foster relationships, mentorships, and networks required by all practising and upcoming neuroprofessionals in the region. Many papers presented at the WFNR conference were selected for inclusion in this issue.

Studies included in this issue have used a range of methodologies to examine the behavioural and cognitive effects of cerebrovascular diseases. The collection of studies also highlights important multidimensional, multidisciplinary, and interdisciplinary collaborations. Such efforts are vital if the rising burden of behavioural and cognitive sequelae of cerebrovascular diseases is to be curtailed. We have included original researches, review papers, and an important meta-analysis. In addition, we have included studies highlighting the burden of behavioural and cognitive effects of cerebrovascular diseases. One study introduced a new context-specific instrument to assess depression after stroke, and another assessed the efficacy of a psychosocial intervention for poststroke depression.

In highlighting the burden of behavioural and cognitive effects of cerebrovascular diseases, A. Ojagbemi et al. used random effect meta-analysis and determined the prevalence and most commonly associated factors with poststroke depression in the sub-Saharan Africa. The study reports a prevalence of poststroke depression that overlaps with findings from high-income countries. Also, A. Blane et al. examined cognitive impairment and simulated driving abilities in stroke survivors using a parallel case and control methodology. They report that despite having more cognitive impairment, being a stroke survivor does not preclude safe (simulated) driving.

Cognitive impairment in stroke survivors with aphasia, as well as central poststroke pain, was also examined by different research groups using cross-sectional methodologies. The study led by C.V. Marinelli highlighted the importance of extensive cognitive examination in patients with poststroke aphasia, while the work led by A. H. Bashir found that central poststroke pain is an important phenomenon in the early poststroke period in Africans. A. Ojagbemi et al. examined the validity of a new stroke-specific screening tool for depression using the Hospital Anxiety and Depression Scale as a suitable criterion. O. Olukolade and H. O. Osinowo used a three-group randomised trial to examine pre- and posttest scores on the Beck Depression Inventory and determined the efficacy of cognitive rehabilitation therapy for poststroke depression. In line with the findings of a meaningful efficacy for cognitive rehabilitation therapy in the study, the authors suggested the integration of the psychosocial intervention as part of the care strategy for poststroke depression.

Three important narrative reviews in this issue examined the pathophysiology of behavioural and cognitive sequelae of cerebrovascular diseases as well as autism as seen after traumatic brain injuries in some children.

There are still important gaps in the literature on the behavioural and cognitive effects of cerebrovascular diseases that are not covered in this issue. Information on emerging environmental and genomic risk factors for behavioural and cognitive complications of cerebrovascular diseases in LMICs is currently lacking. Also, the need to develop tailored and culturally sensitive intervention to stem the rising burden of these complications is pressing in the region. Resources and technologies required for such endeavours are not generally available in many LMICs. However, ongoing investigations by our group and others will provide important context-based information in these relatively novel areas in the near future.

*Mayowa Owolabi* *Akin Ojagbemi* *Raj Kalaria* *Fred Stephen Sarfo* *Rufus Akinyemi*
